# Impact of an Extreme Winter Storm Event on the Coagulation/Flocculation Processes in a Prototype Surface Water Treatment Plant: Causes and Mitigating Measures

**DOI:** 10.3390/ijerph16152808

**Published:** 2019-08-06

**Authors:** Fuguo Qiu, Huadong Lv, Xiao Zhao, Dongye Zhao

**Affiliations:** 1 Key Laboratory of Urban Stormwater System and Water Environment (The Ministry of Education of China), Beijing University of Civil Engineering and Architecture, Beijing 100044, China; 2 Environmental Engineering Program, Department of Civil Engineering, 238 Harbert Engineering Center, Auburn University, Auburn, AL 36849, USA; 3 College of Water Resources & Civil Engineering, China Agricultural University, Beijing 100083, China

**Keywords:** coagulation, flocculation, turbidity, natural organic matter (NOM), stormwater, polyaluminum chloride

## Abstract

Climate change has often caused failure in water treatment operations. In this study, we report a real case study at a major surface water treatment plant in Alabama, USA. Following a severe winter storm, the effluent water turbidity surged to >15.00 Nephelometric Turbidity Units (NTU), far exceeding the 0.30 NTU standard. As a result, the plant operation had to be shut down for three days, causing millions of dollars of losses and affecting tens of thousands of people. Systematic jar tests were carried out with sediment samples from 22 upstream locations. The coagulation and settleability of sediment particles were tested under simulated storm weather conditions, i.e., low temperature (7 °C) and in the presence of various types and concentrations of natural organic matter (NOM) that was extracted from the local sediments. Experimental results proved that elevated NOM (6.14 mg·L^−1^ as Total Organic Carbon, TOC) in raw water was the root cause for the failure of the plant while the low temperature played a minor but significant role. Pre-oxidation with permanganate and/or elevated coagulant dosage were found effective to remove TOC in raw water and to prevent similar treatment failure. Moreover, we recommend that chemical dosages should be adjusted based on the TOC level in raw water, and a reference dosage of 0.29 kg-NaMnO_4_/kg-TOC and 19 kg- polyaluminum chloride (PACl) /kg-TOC would be appropriate to cope with future storm water impacts. To facilitate timely adjustment of the chemical dosages, the real time key water quality parameters should be monitored, such as turbidity, TOC, Ultraviolet (UV) absorbance, pH, and color. The findings can guide other treatment operators to deal with shock changes in the raw water quality resulting from severe weather or other operating conditions.

## 1. Introduction

Over the last several decades, extreme weather events associated with climate change have been gaining a surging momentum in frequency, latitude and impact [1,2,3]. Accordingly, impacts of climate change on water treatment facilities have elicited growing attention [4]. For example, safe drinking water supply has been frequently challenged by extreme weather events such as storms and abrupt temperature fluctuation, which often cause sharp variation in raw water quality such as elevated turbidity, color, and the incursion of natural organic matter (NOM) and colloidal minerals into the source water [5]. Such abrupt changes in raw water characteristics and environmental conditions (e.g., temperature) often result in failure to meet the treatment goals [6,7]. A number of US water utilities have experienced operational difficulties connected with shock loading of dissolved organic carbon (DOC) during the autumn and winter periods. For example, Sharp et al. reported that seasonally elevated DOC in raw water increased the coagulant demand and produced more disinfection byproducts (DBPs) [8]. Because most water treatment facilities are designed based on historical meteorological data without considering such extreme weather conditions, the impacted water treatment plants often fail to take proper response measures to mitigate the impacts. To help water utilities develop such adaptation/response measures, there is an urgent need to understand the associated cause and effects, and to develop science-based knowledge and toolsets geared toward rapid diagnosis of event causes and proper remediation measures.

The Decatur Water Treatment Plant (DWTP), located in Decatur, Alabama, USA, supplies an average of 30 million gallons a day (MGD) (1.14 × 10^5^ m^3^/day) with a peak design capacity of 68 MGD (2.57 × 10^5^ m^3^/day) to a rather populated and commercially active area in northern Alabama. The plant obtains its source water from the Tennessee River, which is ~652 miles (1049 km) long and serves as the drinking water source for three states (Tennessee, Alabama, and Mississippi) with an average flow of 70,575 ft^3^/s (1998 m^3^/s). The treatment train consists of pre-oxidation of DOM with chlorine and sodium permanganate, coagulation with polyaluminum chloride (PACl), sedimentation, filtration, and disinfection with chlorine.

On 1 January 2011, a severe winter storm hit the Tennessee Valley with a record temperature drop from 21 °C to 7 °C overnight. The recorded overall rainfall amount was 3.8 inches (9.65 cm) and the weather history is provided in Figure S1 in the Supporting Information (SI). The heavy rain started at 9:00 pm on 31 December 2010, then it turned to light rain at 9:53, followed by rainy, cloudy, strong thunderstorms overnight. The extreme weather event caused rather unusual raw water quality changes, and thus failure of the treatment process. According to the record (Figure S2 in SI), the effluent water turbidity reached >15.00 Nephelometric Turbidity Units (NTU), which far exceeded the 0.30 NTU limit, despite elevating the PACl dosage up to 86 mg·L^−1^ (which is more than double the normal coagulant dosage, ~30 mg·L^−1^). Consequently, the water treatment plant was forced to shut down for three days until 4 January, when the raw water turbidity went back to the normal level. The 3 day shutdown in water supply resulted in millions of dollars of losses to the department stores and caused substantial chaos and panic of the local residents. In addition to the turbidity surge, the water color was elevated from 10–20 Color Units (CU) before the event to >127 CU during the event; the concentration of iron also raised from <0.3 mg·L^−1^ to 4.10 mg·L^−1^, though the alkalinity and pH remained about the same.

A number of factors are known to affect the coagulation/flocculation processes, including type and concentration of the suspended solids (SS), type and concentration of DOM, temperature, and combinations of these factors [7,9]. However, in practical operations, rapid and accurate diagnosis of key affecting factors is essential to facilitate rapid and effective response measures and to prevent treatment failure. As our knowledge in this area continues to evolve, it is of great practical value to develop a generic, science-based diagnosis-response guide [10]. As the incident occurred at DWTP has been the norm rather than exception, a case study of the underlying causes and the knowledge derived would be of general significance toward developing an integrated knowledge base. As such, the goal of this study was to improve our understanding of the effects of SS (turbidity), DOM, temperature, and combinations thereof on the coagulation/flocculation processes in the context of extreme weather events, and to propose the optimum response measures. The specific objectives were to (1) investigate the causes for the failure in the turbidity removal at DWTP following the storm, and (2) develop a generic knowledge base for plant operators to take emergency response measures to prevent costly disruption of the water treatment operations.

## 2. Materials and Methods

### 2.1. Raw Water and Sediments

About 200 gallons (757.08 L) of water sample was obtained from the Tennessee River near the water intake on a typical spring day. Table 1 gives the key water quality parameters for the river water (information about concentrations of other main elements can be found in Table S1 in the SI). Under normal conditions, the contents of heavy metals and organic matter were very low, whereas alkalinity and hardness were moderate.

**Figure 1:**
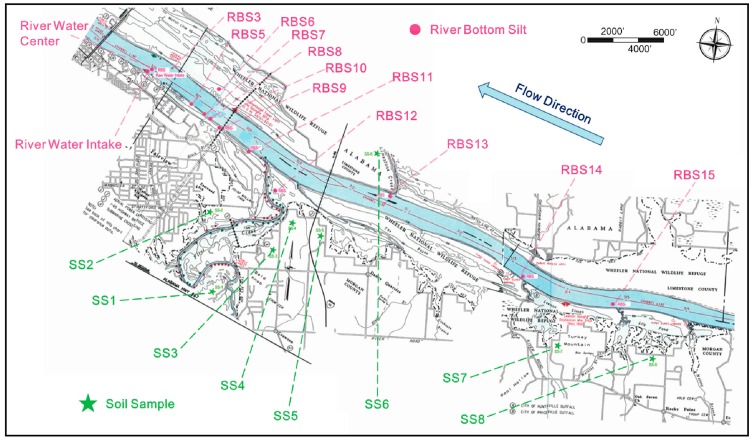
Sampling sites for various sediment samples along Tennessee River.

Because storm water could impact the raw water characteristics through erosion and resuspension of the river bank and bottom sediments, a total of 22 river bottom sediment samples were obtained along the upstream of the river. Figure 1 shows the sampling locations.

The sediments were used to simulate the turbidity surge associated with stormwater runoff. The sediment samples included 1 river bottom silt sample at the water intake (RWI), 1 river bottom silt at the river center near the intake (RWC), 12 upstream bottom silt samples (River bottom silt, RBS3 and RBS5 through RBS15), and 8 surface soil samples (SS1–SS8). The sampling covered a distance of ~15.24 km along the river. All samples were taken within two months after the storm events, and thus, the changes in their physical/chemical characteristics were considered negligible. The raw silts were directly mixed with about 15 L of raw river water to stimulate the raw stormwater with a turbidity of about 70.00 NTU.

### 2.2. Chemical Reagents

Three National Science Foundation (NSF)-certified coagulants, PACl, alum, and ferric chloride, which were used by DWTP, were adopted to test the effectiveness for removal of the turbidity in the raw water samples prepared with the various sediments. PACl (Trade Designation: EC-309S) contains 16% Al_2_O_3_ and with a specific gravity of 1.32; alum (Trade Designation: EC-109) is a solution of 49% dry aluminum sulfate with a 10% Al_2_O_3_ content and a specific gravity of 1.33; and ferric chloride (Trade Designation: CPF-4109) is a solution containing 39% FeCl_3_. In addition, the effect of pre-oxidation on the coagulation/flocculation was tested using two oxidizing agents (a solution of 20% NaMnO_4_ and a NaOCl solution with 5% free chlorine), which were also employed by DWTP.

### 2.3. DOM Extraction

To test the DOM effect on turbidity removal, more concentrated DOM was obtained by extracting the sediment samples RBS-9 and RBS-14. The two sediments were mixed first at a 1:1 mass ratio. The extraction was performed by mixing 1 kg of the mixed wet sediments with five liters of the river water for 24 h and stirred continually, and then settled for 12 h. Then, the supernatant was centrifuged at 6000 rpm for 15 min, and then filtered with 0.45 μm membrane. The resulting filtrate had a DOM level of 18 mg total organic carbon (TOC)·L^−1^, a turbidity of 90.00 NTU, and a pH level of 6.9.

### 2.4. Jar Tests on Turbidity Removal

The standard jar tests for testing the effectiveness of turbidity removal via coagulation, flocculation, and sedimentation were carried out. Two parallel sets of jar testers (Phipps and Bird Sttier 7790-400 and Phipps and Bird PB-700, Richmond, VA, USA) were employed in parallel under identical conditions, each set consisting of 6 paddle stirrers, jars, and 1.5 L glass beakers. The test protocol from DWTP was used, with the following mixing and settling procedures: Sequential mixing at 70 rpm for 1 min, 180 rpm for 30 s, 40 rpm for 15 min, 30 rpm for 15 min, 20 rpm for 15 min, and finally 15 rpm for 25 min; and then settling for 21 min under gravity. At the end of each settling period, ~50 mL of the supernatant was sampled for turbidity analysis using a pipette submerged at ~5 cm below the water surface.

First, a series of screening tests were conducted to probe settling behavior of the 22 sediment samples taken from various locations because these fine particles and the associated substances can be entrained into the source water by stormwater runoff. Known amounts (100–200 g) of each sediment were mixed with 15 L of the river water, stirred at 20 rpm for 5 min and settled for 1 min, then the suspension turbidity was measured. The sediment mass was adjusted until an initial turbidity of 70.00 NTU was achieved for all cases to facilitate a fair comparison of their settling behaviors. The temperature was kept at 21 °C. Based on the water utility’s operating data, the treatment was initiated at a coagulant PACl (EC-309S) dosage of 30 and 45 mg·L^−1^ (as bulk coagulant) from a stock solution of 60 g·L^−1^. The initial pH and alkalinity of the raw water were 7.2–7.5 and 65–70 mg·L^−1^ as CaCO_3_, respectively. All tests were conducted in duplicates to ensure data quality. Results were plotted as mean of duplicates and error bars were calculated as standard deviation to indicate data reproducibility.

Based on the screening tests, RWI and RBS12 were found to be the two most recalcitrant sediments to the coagulation process, and thus were tested further.

### 2.5. Effects of Temperature

As was the case for DWTP, low temperature appeared to play an important role in the process failure. The effect of temperature was tested by comparing the turbidity removal at 21 °C and 7 °C using the sediment sample RWI (the raw water temperature during the DWTP January event was 7 °C). The low water temperature was maintained by placing the beakers in cold-water bath with ice bags. The tests were carried out at three coagulant (EC-309S) dosages, i.e., 30, 60, and 90 mg·L^−1^. The initial pH and alkalinity were 7.47 and 68 mg·L^−1^ as CaCO_3_, respectively.

### 2.6. Effects of DOM

Effects of DOM on the coagulation process were tested using the DOM extracted from the local sediments. The default DOM level in the mixture of RWI and the river water was 3.4 mg·L^−1^ as TOC. For comparison, the TOC level was increased to 4.8, 6.14, and 9.28 mg·L^−1^ by adding the extracted DOM. The initial turbidity was kept at 72.00 NTU in all cases. The coagulant PACl (EC-309S) dosage was varied at 30, 60, and 90 mg·L^−1^. The suspension pH and alkalinity were 7.47 and 70 mg·L^−1^, as CaCO_3_, respectively. To probe the combined effects of temperature and DOM, the experiments were carried out at 7 and 21 °C, respectively.

### 2.7. Pre-Coagulation DOM Removal and Uses of Alternative Coagulants

Based on the above tests, the abrupt rise in DOM was identified as one of the culprits that crippled the coagulation process at DWTP. To facilitate development of rapid response measures, enhanced oxidation of DOM before the coagulation unit and its effect on turbidity removal were investigated. Two common water-treatment oxidants were tested, namely, a NaOCl solution with 5% free Cl_2_ and a 20 wt% NaMnO_4_ solution. The batch oxidation tests were carried out with the same glass beakers under the following conditions: initial TOC = 6.14 mg·L^−1^, initial turbidity = 70.00 NTU temperature = 7 °C, initial pH = 7.55, alkalinity = 70 mg·L^−1^ as CaCO_3_, and stirring rate = 50 rpm. The reaction was followed for 30 min, then the treated water was tested through the jar test procedure.

In addition, the effectiveness of two other commonly used coagulants (Alum and FeCl_3_) were tested and compared with PACl. The coagulant dosage was varied from 15 to 480 mg·L^−1^, with an initial pH and alkalinity of 7.47 and 67 mg·L^−1^ as CaCO_3_, respectively.

### 2.8. Analytical Methods

Water turbidity was determined using an HACH 2100N Turbidity Meter (detection limit = 0.001 NTU). TOC was measured with a Shimadzu TOC-VCPN analyzer (detection limit = 50 μg·L^−1^). The effect of the coagulants on TOC analysis was found to be negligible. Water alkalinity was obtained per the acid titration technique, and water hardness was measured via the EDTA Titrimetric Method. UV-vis absorption spectra were obtained to determine the characteristics of the DOM using a Hewlett Packard 8453 UV-Visible Spectrophotometer (Hewlett Packard, Waldbronn, Germany) at a wavelength of 254 nm.

## 3. Results and Discussion

### 3.1. Coagulation and Sedimentation of Various Sediment Particles

Table 2 presents the turbidity removal extents of the 22 sediment samples. The residual turbidity ranged from 0.10 to 0.32 NTU at the PACl dosage of 30 mg·L^−1^, and it was reduced to <0.22 NTU when the coagulant dosage was increased to 45 mg·L^−1^. Experimental results indicated that PACl was an effective coagulant to remove the turbidity produced by these sediment samples under normal conditions. The final pH of the treated water was in the range of pH 6.95 to 7.20 in all cases, indicating that the raw water had sufficient alkalinity to buffer the water pH.

The results also indicate that the residual turbidity due to the RWI sediment was the highest, followed by that of RBS12. As such, RWI and RBS12 were tested in more detail. Control tests (without coagulant but under otherwise identical conditions) showed that the residual turbidities with RWI and RBS12 were 13.8 and 10.9, respectively. Therebefore, the coagulant at the dosages used by DWTP was able to effectively cope with the turbidity under normal weather conditions, i.e., the plant failure was not caused by the upstream soil/sediments themselves, and other environmental factors should be investigated.

### 3.2. Effects of Temperature on Turbidity Removal

**Figure 2:**
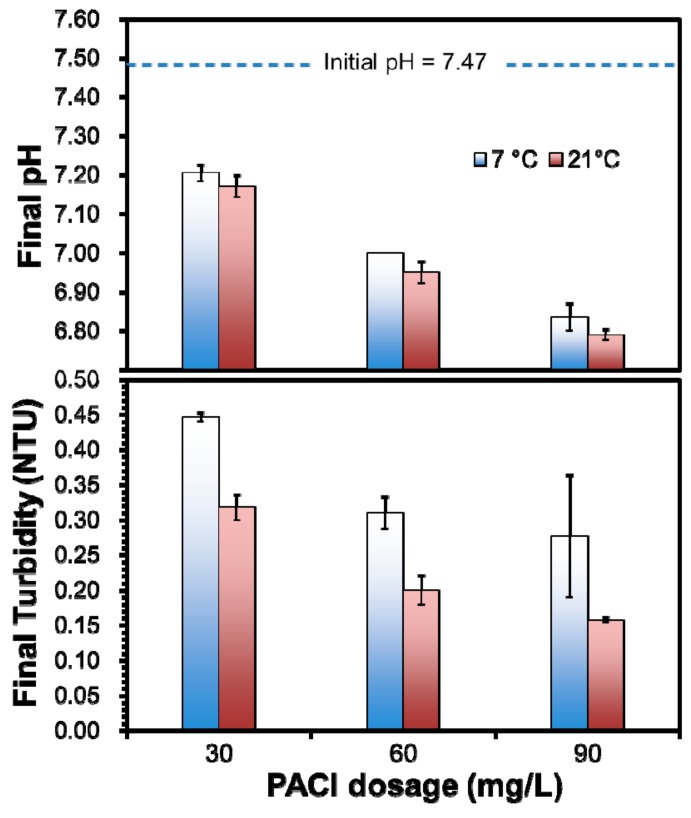
Final pH and turbidity values at three polyaluminum chloride (PACl) dosages and at 7 °C and 21 °C. (Initial pH = 7.47, initial turbidity = 70.00 NTU; data plotted as mean of duplicates and error bars calculated as standard deviation to indicate data reproducibility).

Based on the screening test results, settling of the RWI sediment was further tested at the winter storm temperature (7 °C) and normal temperature (21 °C). The turbidity, pH, and TOC in stimulated raw water was 71.00 NTU, 7.47, and 3.40 mg·L^−1^, respectively. Figure 2 shows that while increasing the coagulant dosage from 30 to 90 mg·L^−1^ progressively increased the turbidity removal, temperature did show a notable effect on the final turbidity. For instance, at the coagulant dosage of 30 mg·L^−1^, the final turbidity at 7 °C was 27% higher than at 21 °C. Moreover, at the coagulant dosage of 60 mg L^−1^ or higher, the turbidity was able to meet the 0.30 NTU limit at the normal temperature, but failed at 7 °C.

Temperature influences coagulation in two ways. First, water temperature slows down the particle collision and chemical reaction rates, resulting in poorer hydrolysis process of coagulants. Second, the relevant physical properties of water, such as viscosity and density, vary with the change of temperature, which in turn can affect coagulation and flocculation behavior. Temperature influences aluminum hydrolysis and solubility based on the hydrolysis constants and the ion product of water, and this effect can be more profound when the hydrolysis occurs in situ [11,12]. However, Morris et al. [13] and Xiao et al. [12] asserted that low temperature conditions may not significantly inhibit the rate of the hydrolysis of aluminum. Thus, the decrease of turbidity removal efficacy under low temperature conditions could be related to fundamental changes in the floc characteristics. For instance, we found that flocs formed at 7 °C (with a diameter of ~2 mm) were smaller than those formed at 21 °C (with a diameter of ~4–5 mm).

Figure 2 shows that the difference in the final pH for the two temperature levels was less than 0.4 pH in all cases, confirming that the effect of temperature on the alum hydrolysis played a minor role. As expected, increasing the coagulant dosage progressively decreased the pH due to the OH^−^ consumption by the PACl hydrolysis, and the pH drop profiles appeared comparable for the two temperatures.

In addition, the slower reaction and flocculation rates at the lower temperature and insufficient hydraulic residence time for flocculation also played a role in the treatment failure. According to the DWTP operating record, five out of their total 14 flocculators were out during the winter storm, which resulted in decreased mixing and flocculation time in the remaining nine flocculators. To test the effects of the shortened flocculation time, we conducted experiments with a much shorter flocculation time (70 rpm for 0.5 min, 180 rpm for 15 s, 40 rpm for 7.5 min, 30 rpm for 7.5 min, 20 rpm for 7.5 min, and finally 15 rpm for 12.5 min; and then settling for 21 min under gravity) with a coagulant dose of 30 mg·L^−1^. At 21 °C, the final turbidity was 0.34 NTU, which is comparable to that (0.32 NTU) under normal flocculation conditions; while at 7 °C, the final turbidity was increased from 0.45 (normal flocculation time) to 0.56 NTU (shortened flocculation time). This observation indicates that shutting off the five flocculators only modestly affected the effluent turbidity, which is consistent with the plant operating record. The shortened flocculation time was able to meet the required effluent water quality under normal weather conditions, but it became insufficient for smaller particles to aggregate into larger flocs under the extreme weather conditions. Morris et al. [13] and Xiao et al. [12] found that increasing floc-growth time was able to counterbalance the consequence with the smaller flocs at low temperatures and improve turbidity removal efficiency.

### 3.3. Effects of NOM on Coagulation/Flocculation

**Figure 3:**
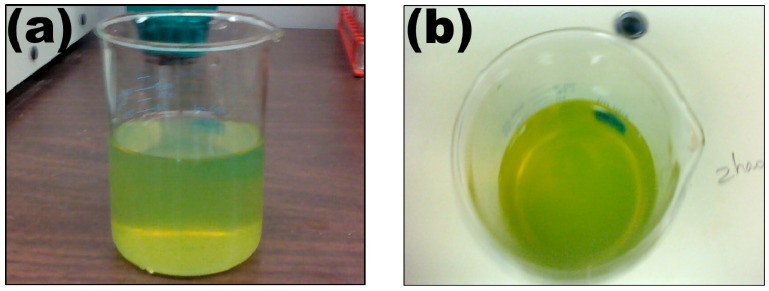
Natural organic matter (NOM) solutions extracted from RBS9 and RBS14 sediments (**a**) side view (**b**) top view.

NOM or DOM is ubiquitous in natural waters because of interactions between the hydrosphere, biosphere, and geosphere. Organic matter found in natural waters mainly consists of humic substances, i.e., humic acid, fulvic acid, and humins. Typically, humic substances account for 71.4–82.5% of total organic carbon content in groundwater and surface waters [14,15]. Humic substances are often associated with yellow and brown colors in raw water. Consequently, water color can often serve as an indicator of NOM presence. The raw water color at DWTP increased from the normal range of 10–20 CU to a maximum of 127 CU during the winter storm, indicating a much elevated NOM content in the river water owing to the storm water runoff. The plant operating data indicated that at 7:00 a.m., 2 January 2011, all filters but 1–10 were shut down. The water was green on top of the filters and the raw water turbidity was in the 70s, confirming the high color level and organic content. Figure 3 shows the color of two NOM solutions extracted from the RBS9 and RBS14 sediments. The solution color is consistent with that recorded in the DWTP operating log book.

**Figure 4:**
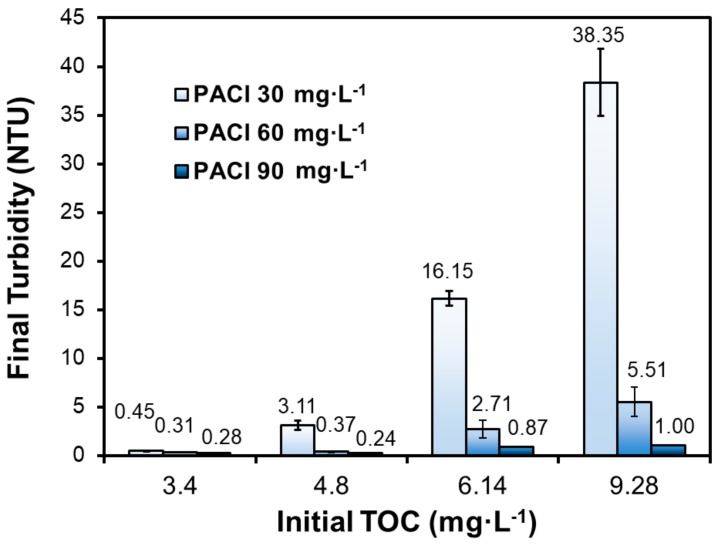
Final turbidity at four initial TOC levels with three PACl dosages at 7 °C. (Initial pH = 7.47, initial turbidity = 70.00 NTU; data plotted as mean of duplicates and error bars calculated as standard deviation to indicate data reproducibility).

Figure 4 compares the final turbidity in the presence of various levels of the extracted DOM at 7 °C. The residual turbidity increased remarkably with the elevated TOC level at all three PACl dosages of 30, 60, and 90 mg·L^−1^. NOM can be adsorbed or coated on inorganic particles, and thus stabilize the particles though electrostatic and/or steric stabilization mechanisms [16]. For instance, Wilkinson et al. [17] reported that fulvic substances can stabilize inorganic colloids and particles by modification of their surface potential, and Kretzschmar et al. [18] observed that a humic acid at up to 2 mg·L^−1^ was able to cause a more negative zeta-potential of kaolin, resulting in greater colloidal stability of the kaolinite suspension.

Evidently, with even the lowest DOM level of 3.4 mg/L as TOC, the normal coagulant (EC-309S) dosage of 30–60 mg·L^−1^ was insufficient to treat the water to meet the required turbidity. The water turbidity surged remarkably with increasing NOM, reaching as high as 38.35 NTU in the presence of 9.28 mg·L^−1^ TOC and at the coagulant dosage of 30 mg·L^−1^. The results provide strong evidence that elevated NOM level, combined with the low temperature, was an important root cause of the plant failure. Moreover, the results showed that at the initial TOC of 6.14 mg·L^−1^, the residual turbidity was 16.15 NTU at the PACl dosage of 30 mg·L^−1^, which was in agreement with the observed plant data during the event. Interestingly, increasing the coagulant dosage to 90 mg·L^−1^ was able to suppress the residual turbidity to ≤1.00 NTU in all cases and to meet the 0.30 NTU threshold when the NOM was ≤4.8 mg·L^−1^.

The results indicate that raw water TOC level is a key factor in turbidity removal, and a very practical and effective measure to cope with the sudden rise of influent TOC is to increase the coagulant dosage.

**Figure 5:**
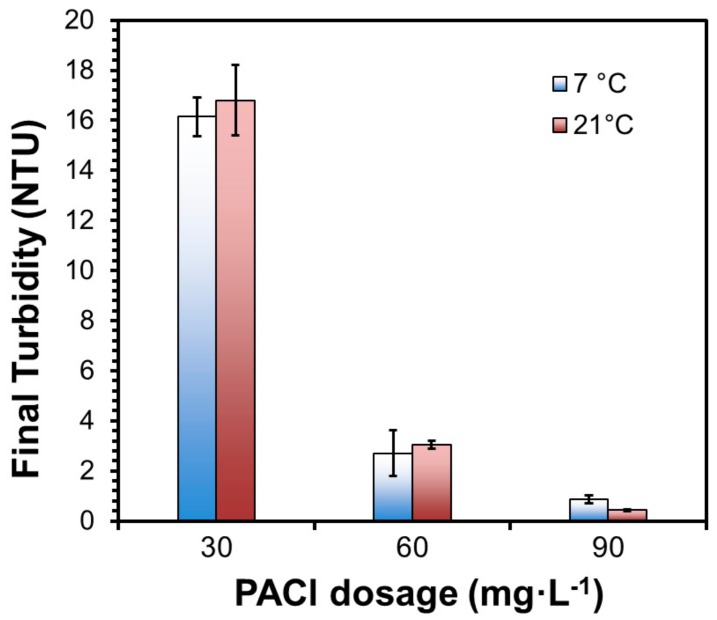
Turbidity removal at 7 °C and 21 °C with three PACl dosages. (Initial pH = 7.47, initial turbidity = 70.00 NTU, initial TOC = 6.14 mg·L^−1^; data plotted as mean of duplicates and error bars calculated as standard deviation to indicate data reproducibility).

To probe the relative importance of temperature and NOM, turbidity removal was tested at two different temperatures and in the presence of 6.14 mg·L^−1^ as TOC of NOM. Figure 5 shows that the difference in turbidity removal at 7 °C and 21 °C was statistically insignificant at all three coagulant dosages. Taken together, the results in Figure 2 and Figure 5 show that the temperature effect became less important when the NOM level was high, in other words, the sudden rise in NOM is more likely the culprit for the process failure. In addition, Figure 5 confirms that increasing coagulant dosage is a simple but effective way to cope with the shock change in influent water NOM.

Our results also showed that the coagulation-flocculation-sedimentation process removed ~20% TOC (initial TOC = 6.14 mg·L^−1^) at all three dosages and temperature had an insignificant effect on the TOC removal, indicating that only a fixed fraction of the NOM was absorbable to the coagulated flocs. This is reasonable because the NOM was water-extracted from the field sediment and represents the fraction of NOM that is more prone to storm water dissolution. Combining this observation and the results in Figure 5 reveals that while the coagulant was rather effective to remove the turbidity, it only removed a fixed fraction of the NOM, which again indicates the existence of only limited functional groups of the NOM that can bind with the solid particles. The raw water had sufficient alkalinity to buffer the water pH due to the increased coagulant dose. For instance, when the coagulant dosage was raised from 30 to 90 mg·L^−1^, the pH dropped from 7.23 to 7.03, and the alkalinity consumption was increased from 8.8 to 15.5 mg·L^−1^ as CaCO_3_ (initial TOC = 6.14 mg·L^−1^, temperature = 7 °C). Again, temperature effects on the pH and alkalinity consumption were insignificant.

NOM in natural waters can include both hydrophilic and hydrophobic portions. The hydrophilic portion is composed mainly of aliphatic carbon and nitrogenous compounds including carboxylic acids, carbohydrates, and proteins, whereas hydrophobic NOM primarily consists of humic and fulvic acids (humic substances) containing aromatic rings, phenolic structures, and conjugated double bonds. At pH values above 4, humic substances can be considered as natural anionic polyelectrolytes [19]. It has been well known that coagulation preferentially removes higher molecular weight and more hydrophobic NOM, and conversely, larger and more hydrophobic NOMs are less prone to dissolution by storm water. Hence, lower molecular weight and more hydrophilic NOMs are more likely to be washed off the upstream soil/sediment, enter into the raw water, and escape the coagulation process [20]. For the same reason, this type of NOM is more likely what upset the coagulation process.

Edzwald et al. [21] conceived the concept of Specific UV Absorbance (SUVA) and found SUVA correlates well with the aromaticity and the hydrophobicity of NOM, where a higher SUVA suggests a better NOM treatability by coagulation. Table 3 gives the SUVA guidelines proposed by Edzwald et al. [21].

**Figure 6:**
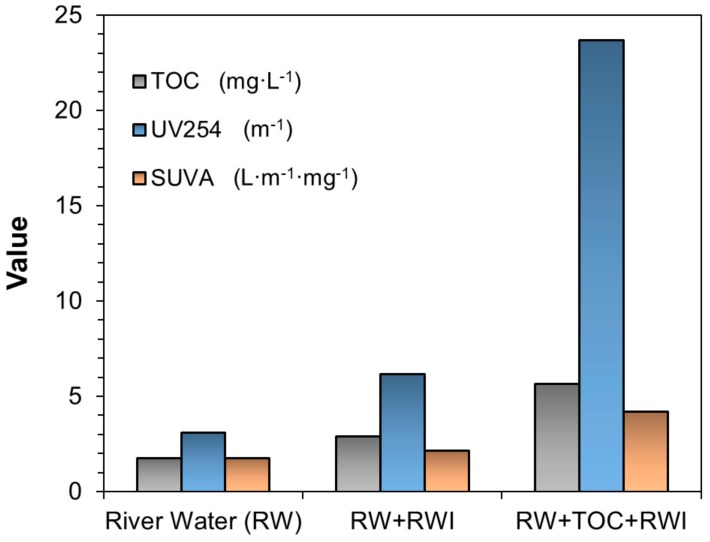
TOC, UV254, and Specific UV Absorbance (SUVA) values of three simulated raw water samples. (For river water conditions please refer to Table 1, for RW + RWI and RW + TOC + RWI, initial pH ≈ 7.50, turbidity = 70.00 NTU, temperature = 21 °C).

Figure 6 presents the SUVA values along with the TOC and UV254 values for the plain river water, and the river water modified with the addition of RWI sediment and TOC. The SUVA value of the normal river water was 1.77, so the NOM is comprised of mostly non-humic matter with low hydrophobicity and low molecular weight, and it has little influence on coagulation, and thus the normal DWTP coagulant dosage at 30–60 mg·L^−1^ was able to meet the required turbidity removal. The RWI-modified raw water gave a turbidity of 70.00 NTU had a SUVA value of 2.14, suggesting that the NOM exerts more influences on coagulation. However, because the SUVA value is quite close to the threshold of two in Table 3, the NOM effects on coagulation was rather modest, and PACl at the dosage of 30 mg·L^−1^ was able to achieve a ~0.30 NTU residual turbidity, and a TOC removal of 15–26% at 21 °C. Hence, the NOM in RWI stimulated water was also of low hydrophobicity and low molecular weight. In contrast, the SUVA value of the raw water modified with extracted NOM and RWI was 4.19, suggesting NOM dominated the coagulation process. The trend is consistent with the data in Figure 5, which shows the residual turbidity remained at 3.00 NTU even at the coagulant dosage of 60 mg·L^−1^. However, the TOC removal at the PACl dosage range of 30–90 mg·L^−1^ remained at 16–23% at 21 °C, which does not conform to the DOC removal guideline in Table 3, which can be attributed to the different nature of the NOM and indicates only a fixed fraction of the NOM was reactive to the coagulated particles in the DWTP storm water.

### 3.4. Measures to Cope with Turbidity Upset Due to Elevated NOM Shock Loading

It is evident from afore-presented results that controlling the raw water NOM and manipulating the coagulant dosage is the key to prevent the turbidity upset and process failure. To this end, the coagulant dosage should be adjusted based on the TOC level in the raw water.

**Figure 7:**
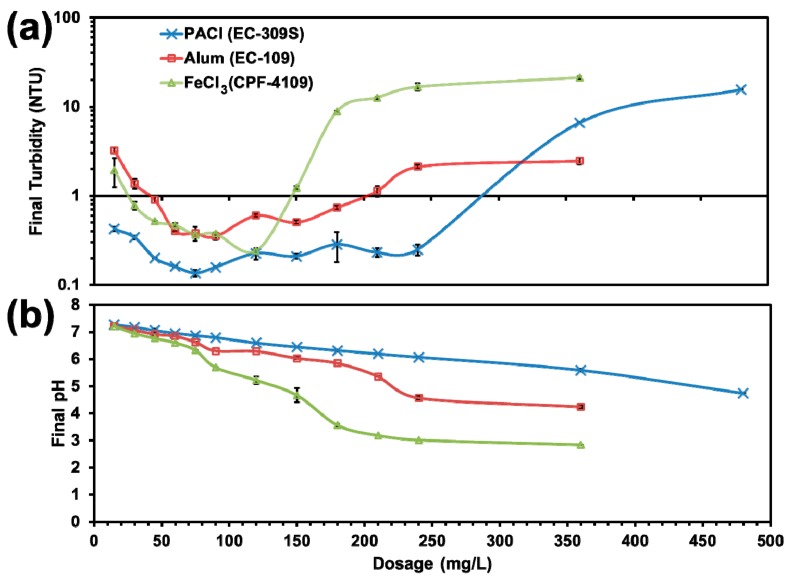
(**a**) Turbidity removal by three coagulants at various dosages; (**b**) pH changes for three coagulants at various doses. (Temperature = 7 °C, initial pH = 7.47, initial turbidity = 70.00 NTU, initial TOC = 6.14 mg·L^−1^; data plotted as mean of duplicates and error bars calculated as standard deviation to indicate data reproducibility).

To find the optimum coagulant and dosage, Figure 7a compares the jar test results using three commonly used coagulants and at a range of coagulant dosages. In general, the results concurred with the classical coagulation curve. As expected, PACl gave the best turbidity removal, whereas the alum and FeCl_3_ showed comparable effectiveness. For all the three coagulants, the optimum dosage was found at around 75 mg·L**^−^**^1^, and no advantage was observed at a higher dosage because of the charge reversal effect that led to the restabilization of the particles. While PACl was able to achieve a final turbidity of <0.20 NTU, Alum and FeCl_3_ only reached a level of 0.40 NTU under the simulated severe weather conditions. However, the superior performance of PACl, which is currently used as the primary coagulant at DWTP, comes with a higher cost. Figure 7b shows PACl results in much less impact on the water pH than alum and FeCl_3_ at various coagulant dosages. Based on the data in Figure 7, at the TOC level of 3.4 mg·L**^−^**^1^ and the optimum coagulant dosage of 75 mg·L**^−^**^1^, a coagulant dosage of 22 kg-PACl/kg-TOC would be recommended, which agrees with the dosage of 19 kg-PACl/kg-TOC derived from Figure 4, where the addition of 90 mg·L**^−^**^1^ of PACl was able to bring the turbidity down to <0.30 NTU in the presence of 4.8 mg·L**^−^**^1^ of TOC.

It is cautioned though that other weather factors should be taken into account such as temperature and storm duration. For instance, a higher temperature and longer raining time may wash off larger and more hydrophobic NOM (of higher SUVA) into the river water, which may interfere more with the coagulation process. The TOC-based coagulant dose is easy to implement with existing installations. To facilitate timely action, an online real-time TOC monitoring system should be available. However, this method bears with a downside of low NOM removal (only ~20%), raising the concern of producing chlorinated DBPs in the disinfection stage.

**Figure 8:**
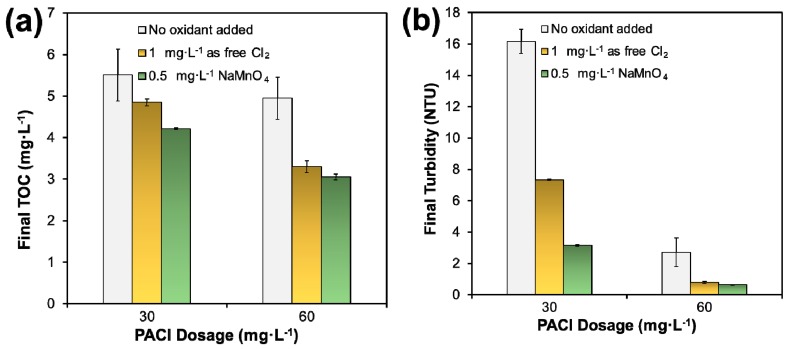
(**a**) TOC removal by NaOCl (1 mg·L^−1^ as free Cl_2_) or NaMnO_4_ before coagulation; and (**b**) Turbidity removal with or without pre-coagulation oxidation of NOM using NaOCl or NaMnO_4_. (Temperature = 7 °C, initial turbidity = 70.00 NTU, pH ≈ 7.50, initial TOC = 6.14 mg·L^−1^; data plotted as mean of duplicates and error bars calculated as standard deviation to indicate data reproducibility).

To avoid DBP production and prevent interfering with coagulation, pre-coagulation oxidation of NOM had been practiced at DWTP, though not on a regular basis and it was not deployed during the winter storm. Figure 8a shows that the pre-oxidation treatment greatly enhanced the removal of TOC in the coagulation process. In particular, at the coagulant dosage of 60 mg·L^−1^, 46% of initial TOC was removed with 1 mg·L^−1^ of free Cl_2_ oxidation and the subsequent coagulation, and over 50% of TOC was removed with 0.5 mg·L^−1^ NaMnO_4_ followed by coagulation, compared to only ~19% without the pre-oxidation treatment. After the pre-treatment, no residual free chlorine and soluble Mn^2+^ were detected, indicating complete consumption of the oxidants by the basic chlorine demand substances such as DOM and reducing compounds in the raw water. Figure 8b indicates that the pre-oxidation of NOM greatly enhanced the coagulation efficiency. For instance, the turbidity removal reached 99% at the PACl dosage of 60 mg·L^−1^ with either of the pre-oxidation treatments. The pre-oxidation may facilitate coagulation in two ways, including oxidation of adsorbed organics and their hydrophilization and eventual desorption led to destabilization of the particles, and catalyzed polymerization of NOM and subsequent adsorption bridging [22,23]. It is also evident that NaMnO_4_ was more efficient at enhancing the turbidity and TOC removal than chlorine. This is consistent with a report by Ma et al. [24] that permanganate was more effective than chlorine for enhancing coagulation of very stable surface waters with relatively high organic matter (5 mg·L^−1^ humic acid). The authors claimed that MnO_2_ produced in situ during permanganate pre-oxidation was able to adsorb NOM and act as nuclei to aid in coagulation by the seeding effect, and the resulting flocs had a bigger size and higher density. Moreover, Liu et al. [25] reported that permanganate improved the removal of both organic particulates and inorganic fine particles.

However, under the simulated storm water conditions, the pre-oxidation + coagulation did not meet the 0.30 NTU threshold even at the 60 mg·L^−1^ dosage level. According to the operation report of DWTP, the treatment train was able to get through a prior storm at a similar level in November 2010 when the maximum dosage for NaMnO_4_ and chlorine was raised to 1.34 and 4.97 mg·L^−1^, respectively (both were applied during the storm time). However, the plant failed at this January storm when the NaMnO_4_ and chlorine dosage was kept at 0.25 and 0.67 mg·L^−1^ when the raw water turbidity began to increase sharply on 1 January 2011.

Increasing free Cl_2_ dosage may not be recommended due to the concern of DBP production, though it is cheaper than permanganate. A viable solution would be to slightly increase the dosage of permanganate (e.g., 1.5 mg·L^−1^) and/or the dosage of PACl (e.g., 75 mg·L^−1^) during the storm event, which can not only satisfy the effluent turbidity, but also mitigate DBP generation with a reasonable cost. In practice, controlling pH and sludge production are also important factors to determine the optimum dosages for coagulants and pre-treatment chemicals. However, under extreme conditions, such as the severe weather in this study, control of the effluent water quality should be considered the first priority.

## 4. Conclusions

With increasingly occurring extreme weather conditions, traditional water treatment plants are confronted with mounting challenges to comply with increasingly stringent regulations. Based on a real-world process failure at a major surface water treatment plant, this study examined the root cause for the failed coagulation/flocculation/sedimentation at DWTP and proposed a practical solution for plant operators to cope with shock changes in the influent water quality parameters (e.g., NOM, turbidity, and temperature). The key findings are summarized as follows:

1. The key factor causing the DWTP turbidity treatment failure was the suddenly elevated NOM associated with some of the upstream sediment that was washed off by the severe winter storm into the river water. The low temperature had some minor adverse effects on the coagulation, but was not the main cause;
2. Based on the plant data and our simulated experimental results, the native NOM at 6.14 mg·L^−1^ as TOC combined with the low temperature (7 °C) caused the sudden turbidity rise in the treated water and the process failure during the January winter storm;
3. Jar tests using normal river water and river water modified with local sediment and DOM indicated that increasing the PACl dosage based on the raw water TOC level is a viable and practically feasible approach to cope with the shock changes. We recommend that the coagulant dosage should be based on the TOC level. As such, an optimal PACl dosage range of 22 kg-PACl/kg-TOC (under normal conditions) to 19 kg-PACl/kg-TOC (during storm time) or 75–90 mg·L^−1^ is recommended;
4. For the DWTP NOM, the coagulation with PACl removed a fixed fraction (~20%) of the initial DOM, regardless of the coagulant dosage, indicating most of the NOM was less adsorbable or more soluble and may easily reach the final disinfection stage raising the concern of DBPs;
5. The addition of chlorine and/or permanganate before coagulation was able to enhance the TOC removal, improve the turbidity removal by coagulation, and thus lower the coagulant demand. In particular, permanganate appears more cost-effective than NaOCl and does not cause DBPs. Consequently, a sample reference dosage of 0.29 kg-NaMnO_4_/kg-TOC and 19 kg-PACl/kg-TOC is recommended to cope with future storm water impacts in either winter or summer time. To facilitate the TOC-based chemical dosage adjustment, the variation of critical raw water quality parameters should be monitored closely, especially when severe weather is expected. The most critical parameters are raw water TOC and turbidity, in addition, SUVA, pH, and color should also be considered to determine the optimum dosage under specific conditions.

The information derived from this work is of general significance and may be referenced by tens of millions of similar treatment plants that are facing similar challenges associated with the worsening climate changes.

## Figures and Tables

**Table 1:** Summary of key parameters in the river water.

Item	Value	Item	Level (mg·L^−1^)
pH	7.52	Total Organic Carbon (TOC)	1.30
Turbidity (NTU)	3.57	Hardness as CaCO_3_	62.30
		Alkalinity as CaCO_3_	69.00
		Ca	18.00
		Mg	5.62

**Table 2:** Final turbidity (mean of duplicate tests) of raw water mixed with 22 sediments with two dosages of PACl (EC-309S), 30 mg·L^−1^ and 45mg·L^−1^ (initial turbidity = 70.00 NTU, pH = 7.47, temperature = 21 °C).

Dosage (mg·L^−1^)	SS1	SS2	SS3	SS4	SS5	SS6	SS7	SS8	RBS3	RBS5	RBS6
30	0.12	0.11	0.14	0.12	0.13	0.21	0.17	0.17	0.14	0.13	0.15
45	0.16	0.10	0.12	0.09	0.12	0.11	0.10	0.18	0.12	0.12	0.10
**Dosage (mg·L^−1^)**	**RBS7**	**RBS8**	**RBS9**	**RBS10**	**RBS11**	**RBS12**	**RBS13**	**RBS14**	**RBS15**	**RWI**	**RWC**
30	0.16	0.14	0.22	0.14	0.14	0.20	0.15	0.10	0.12	0.32	0.17
45	0.15	0.11	0.19	0.13	0.12	0.20	0.14	0.09	0.16	0.21	0.16

Note: SS: Soil sample; RBS: River bottom silt; RWI: River water intake; RWC: River water center.

**Table 3:** Guidelines on the nature of NOM and expected TOC removals.

SUVA	Composition	Coagulation
<2	Mostly non-humics Low hydrophobicity Low molecular weight	NOM has little influence Poor TOC removal
2–4	Mixture of aquatic humics and other NOM Mixture of hydrophobic and hydrophilic NOM Mixture of molecular weights	NOM influences Fair to good TOC removal
>4	Mostly aquatic humics High hydrophobicity High molecular weight	NOM controls Good TOC removal

Note: SUVA: Specific UV Absorbance; NOM: Nature organic matter.

## Supplementary Materials

The following are available online at https://www.mdpi.com/1660-4601/16/15/2808/s1, Figure S1: Weather History of Decatur, AL, USA (a) weather graph, (b) weather history for 31 December 2010 and (c) weather history for 1 January 2011; Figure S2: Data of turbidity of the raw water and the treated water, and the PACl feeding dose for the plant from 28 December 2010 to 5 January 2011. Table S1: Summary of concentrations of main elements in the river water.
