# Supplementary material for: Impact of an Extreme Winter Storm Event on the Coagulation/Flocculation Processes in a Prototype Surface Water Treatment Plant: Causes and Mitigating Measures
Source: Int J Environ Res Public Health. 2019 Aug 6;16(15):2808. doi: 10.3390/ijerph16152808 (PMC6695649; doi:10.3390/ijerph16152808)
Supplement: Supplementary file 1 [file ijerph-16-02808-s001.pdf]

Supporting Information

Impact of an Extreme Winter Storm Event on the Coagulation/Flocculation Processes in a Prototype Surface Water Treatment Plant: Causes and Mitigating Measures

Fuguo Qiu [1,2], Huadong Lv [1], Xiao Zhao [2,3,*] and Dongye Zhao [2,*]

[1] Key Laboratory of Urban Stormwater System and Water Environment (The Ministry of Education of China), Beijing University of Civil Engineering and Architecture, Beijing 100044, China
[2] Environmental Engineering Program, Department of Civil Engineering, 238 Harbert Engineering Center, Auburn University, Auburn, AL 36849, USA
[3] College of Water Resources & Civil Engineering, China Agricultural University, Beijing 100083, China
* Correspondence: xiaozhao88@cau.edu.cn (X.Z.); zhaodon@auburn.edu (D.Z.); Tel.: +86-10-62736533 (X.Z.); +1-334-844-6277 (D.Z.)

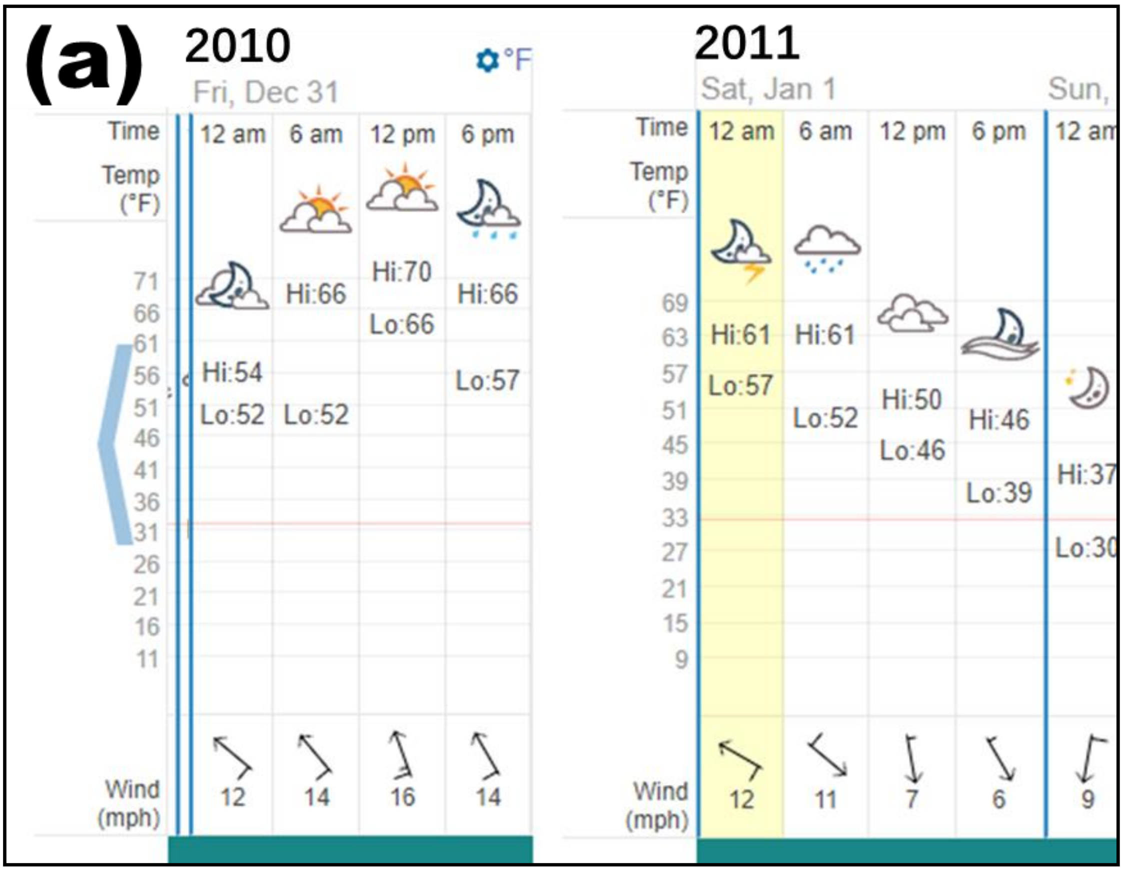
(a)
2010
Fri, Dec 31
°F
Time
12 am
6 am
12 pm
6 pm
Temp (°F)
71
66
61
56
51
46
41
36
31
26
21
16
11
Hi:54
Lo:52
Hi:66
Lo:52
Hi:70
Lo:66
Hi:66
Lo:57
Wind (mph)
12
14
16
14
2011
Sat, Jan 1
Sun,
Time
12 am
6 am
12 pm
6 pm
12 am
Temp (°F)
69
63
57
51
45
39
33
27
21
15
9
Hi:61
Lo:57
Hi:61
Lo:52
Hi:50
Lo:46
Hi:46
Lo:39
Hi:37
Lo:30
Wind (mph)
12
11
7
6
9

(b) City of Decatur Weather History for December 31, 2010

Show weather for: December 31, 2010

Time	Temp	Weather	Wind	Humidity	Barometer	Visibility
12:02 am Fri, Dec 31	54 °F	Light rain. Mostly cloudy.	9 mph	67%	30.03 "Hg	10 mi
12:53 am	54 °F	Overcast.	17 mph	69%	30.02 "Hg	10 mi
1:53 am	52 °F	Overcast.	14 mph	72%	30.03 "Hg	10 mi
2:53 am	52 °F	Overcast.	12 mph	72%	30.02 "Hg	10 mi
3:53 am	51 °F	Overcast.	13 mph	71%	30.01 "Hg	10 mi
4:53 am	52 °F	Overcast.	10 mph	72%	30.03 "Hg	10 mi
5:53 am	52 °F	Overcast.	10 mph	72%	30.04 "Hg	10 mi
6:53 am	52 °F	Overcast.	8 mph	72%	30.06 "Hg	10 mi
7:53 am	54 °F	Overcast.	13 mph	74%	30.07 "Hg	10 mi
8:53 am	55 °F	Overcast.	10 mph	74%	30.09 "Hg	10 mi
9:53 am	58 °F	Mostly cloudy.	12 mph	70%	30.10 "Hg	10 mi
10:53 am	61 °F	Broken clouds.	16 mph	67%	30.07 "Hg	10 mi
11:53 am	66 °F	Partly sunny.	22 mph	61%	30.02 "Hg	10 mi
12:53 pm	68 °F	Partly sunny.	20 mph	57%	29.98 "Hg	10 mi
1:53 pm	69 °F	Partly sunny.	15 mph	55%	29.97 "Hg	10 mi
2:53 pm	69 °F	Partly sunny.	14 mph	55%	29.96 "Hg	10 mi
3:53 pm	68 °F	Overcast.	15 mph	55%	29.95 "Hg	10 mi
4:53 pm	68 °F	Overcast.	16 mph	55%	29.96 "Hg	10 mi
5:53 pm	67 °F	Overcast.	15 mph	59%	29.98 "Hg	10 mi
6:53 pm	67 °F	Overcast.	17 mph	59%	29.98 "Hg	10 mi
7:53 pm	65 °F	Light rain. Mostly cloudy.	17 mph	66%	29.99 "Hg	10 mi
9:03 pm	61 °F	Heavy rain. Fog.	15 mph	83%	30.00 "Hg	4 mi
9:53 pm	59 °F	Light rain. Mostly cloudy.	10 mph	90%	29.99 "Hg	10 mi
10:53 pm	58 °F	Rain. Fog.	15 mph	93%	29.94 "Hg	6 mi
11:02 pm	57 °F	Light rain. Mostly cloudy.	12 mph	94%	29.98 "Hg	8 mi

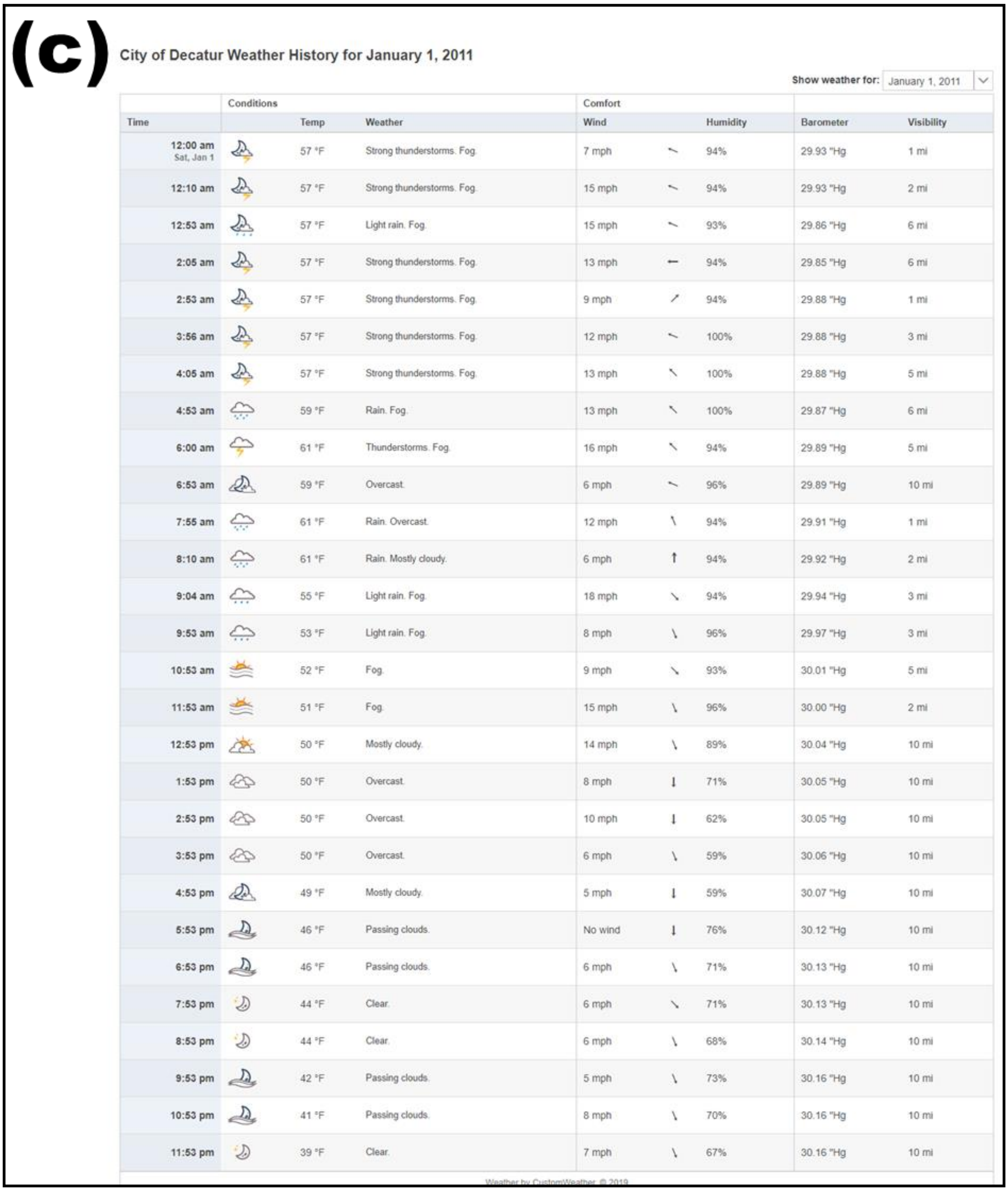

(c) City of Decatur Weather History for January 1, 2011

Show weather for: January 1, 2011

Time	Conditions		Comfort		
	Temp	Weather	Wind	Humidity	Barometer	Visibility

Time	Temp	Weather	Wind	Humidity	Barometer	Visibility
12:00 am Sat, Jan 1	57 °F	Strong thunderstorms. Fog.	7 mph	94%	29.93 "Hg	1 mi
12:10 am	57 °F	Strong thunderstorms. Fog.	15 mph	94%	29.93 "Hg	2 mi
12:53 am	57 °F	Light rain. Fog.	15 mph	93%	29.86 "Hg	6 mi
2:05 am	57 °F	Strong thunderstorms. Fog.	13 mph	94%	29.85 "Hg	6 mi
2:53 am	57 °F	Strong thunderstorms. Fog.	9 mph	94%	29.88 "Hg	1 mi
3:56 am	57 °F	Strong thunderstorms. Fog.	12 mph	100%	29.88 "Hg	3 mi
4:05 am	57 °F	Strong thunderstorms. Fog.	13 mph	100%	29.88 "Hg	5 mi
4:53 am	59 °F	Rain. Fog.	13 mph	100%	29.87 "Hg	6 mi
6:00 am	61 °F	Thunderstorms. Fog.	16 mph	94%	29.89 "Hg	5 mi
6:53 am	59 °F	Overcast.	6 mph	96%	29.89 "Hg	10 mi
7:55 am	61 °F	Rain. Overcast.	12 mph	94%	29.91 "Hg	1 mi
8:10 am	61 °F	Rain. Mostly cloudy.	6 mph	94%	29.92 "Hg	2 mi
9:04 am	55 °F	Light rain. Fog.	18 mph	94%	29.94 "Hg	3 mi
9:53 am	53 °F	Light rain. Fog.	8 mph	96%	29.97 "Hg	3 mi
10:53 am	52 °F	Fog.	9 mph	93%	30.01 "Hg	5 mi
11:53 am	51 °F	Fog.	15 mph	96%	30.00 "Hg	2 mi
12:53 pm	50 °F	Mostly cloudy.	14 mph	89%	30.04 "Hg	10 mi
1:53 pm	50 °F	Overcast.	8 mph	71%	30.05 "Hg	10 mi
2:53 pm	50 °F	Overcast.	10 mph	62%	30.05 "Hg	10 mi
3:53 pm	50 °F	Overcast.	6 mph	59%	30.06 "Hg	10 mi
4:53 pm	49 °F	Mostly cloudy.	5 mph	59%	30.07 "Hg	10 mi
5:53 pm	46 °F	Passing clouds.	No wind	76%	30.12 "Hg	10 mi
6:53 pm	46 °F	Passing clouds.	6 mph	71%	30.13 "Hg	10 mi
7:53 pm	44 °F	Clear.	6 mph	71%	30.13 "Hg	10 mi
8:53 pm	44 °F	Clear.	6 mph	68%	30.14 "Hg	10 mi
9:53 pm	42 °F	Passing clouds.	5 mph	73%	30.16 "Hg	10 mi
10:53 pm	41 °F	Passing clouds.	8 mph	70%	30.16 "Hg	10 mi
11:53 pm	39 °F	Clear.	7 mph	67%	30.16 "Hg	10 mi

Weather by CustomWeather © 2019

Figure S1. Weather History of Decatur, AL, USA **(a)** weather graph, **(b)** weather history for Dec. 31, 2010 and **(c)** weather history for Jan. 1, 2011 (Information from the Website: https://www.timeanddate.com/weather/@7317841/historic?month=11&year=2010).

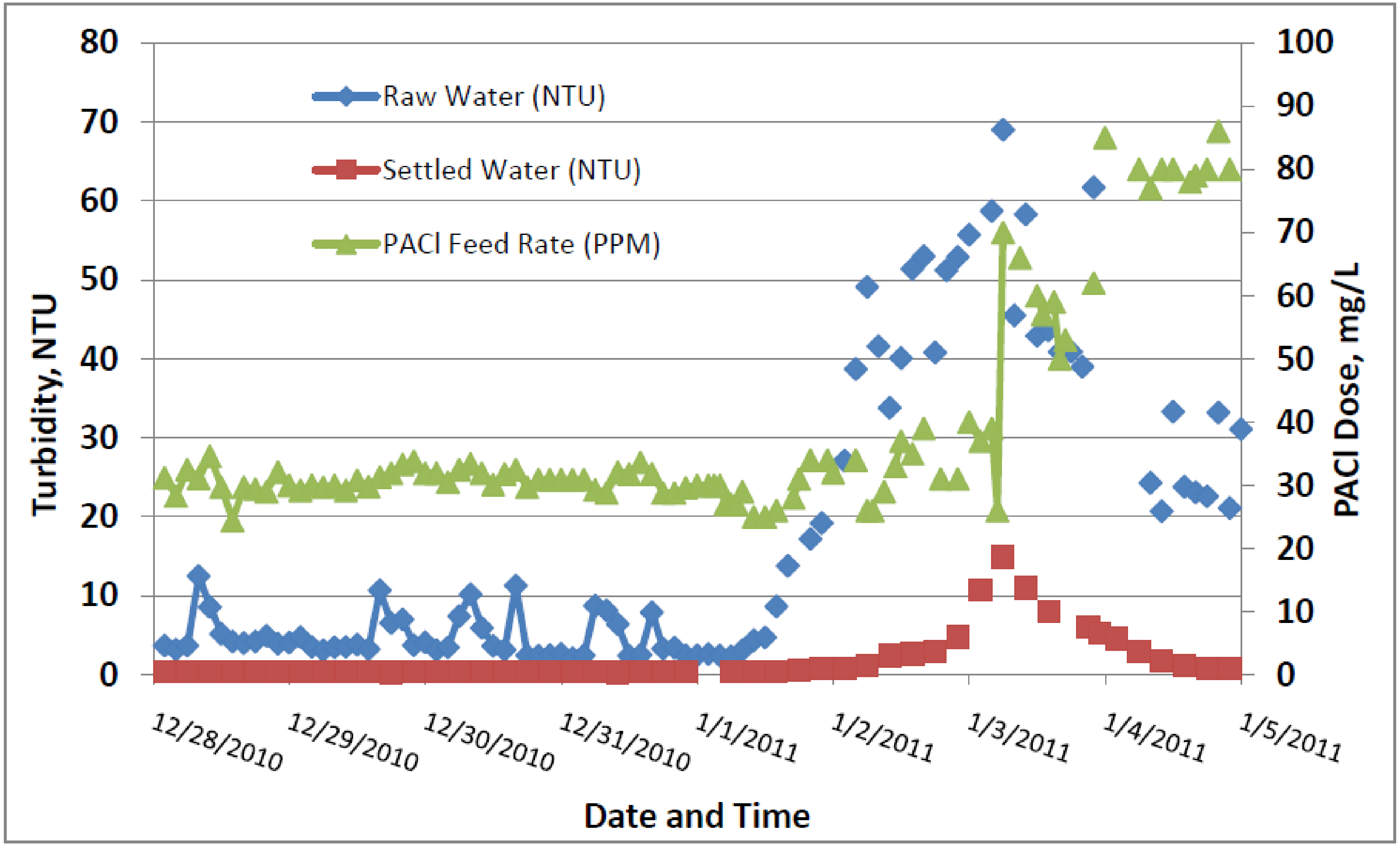

Figure S2. Data of turbidity of the raw water and the treated water, and the PACl feeding dose for the plant from Dec. 28, 2010 to Jan. 5, 2011

Table S1. Summary of concentrations of main elements in the river water.

Item	Level (mg·L^{-1})	Item	Level (mg·L^{-1})	Item	Level (mg·L^{-1})
Na	2.7924	Fe	0.002679	K	1.5050
Si	0.0477	Cu	0.003194	Mn	0.0011
AL	0.0016	Sn	0.003431	P	0.0048
Be	0.0001	B	0.0769	Li	0.0014
Pb	0.0006	Ni	0.031317	Ba	0.0255
Co	0.0002	Sb	0.001864	Mo	0.0080
Ag	0.0003	As	0.00146	Sr	0.0220
Cr	0.0005	V	NA	Cd	0.0001
Zn	NA	Se	NA		
